# Is Aggressive Surgical Palliation of Proximal Bile Duct Cancer With Involvement of Both Main Hepatic Ducts Worthwhile?

**DOI:** 10.1155/1992/91670

**Published:** 1992

**Authors:** Dietmar K. Wilker, Jakob R. Izbicki, Ralf Rohloff, Wolfram-T. Knoefel, Hans Mandelkow, Leonhard Schweiberer

**Affiliations:** 1 Dept. of Surgery University of Munich Germany; 2 Dept. of Radiology and Radiotherapy University of Munich Germany; 3 Dept. of Surgery University of Hansing Germany; 4 Chururgische Klinik Universitat Hamburg Martiuistr. 52 Hamburg 20 D-2000 Germany; 5 der Universität München Nuβbaumstr. 20 München 2 D-8000 Germany

## Abstract

The only curative treatment for proximal bile duct cancer with involvement of both main hepatic ducts is liver transplantation. Most patients do not fulfill the requirements for liver transplantation. Our treatment strategy in appropriate cases is palliative tumor resection and reconstruction of the biliary passage by sutureless bilioenteric anastomosis. We have treated 12 patients, 5 in combination with intraluminal and percutaneous radiotherapy. Our results indicate that this strategy leads to effective palliation in some cases provided that only microscopic residual tumor is left in-situ. Our survival times compare favourably with survival after liver transplantation.


*HPB Surgery*, 1992, Vol. 5, pp. 235–249
Reprints available directly from the publisher
Photocopying permitted by license only

© 1992 Harwood Academic Publishers GmbH
Printed in the United Kingdom

### IS AGGRESSIVE SURGICAL PALLIATION OF PROXIMAL BILE DUCT CANCER WITH INVOLVEMENT OF BOTH MAIN HEPATIC DUCTS WORTHWHILE?

DIETMAR K. WILKER[1], JAKOB R. IZBICKI[1,3], RALF ROHLOFF[2], WOLFRAM-T. KNOEFEL[1,3], HANS MANDELKOW[1] and LEONHARD SCHWEIBERER[1]

*[1]Dept. of Surgery, University of Munich, FRG*
*[2]Dept. of Radiology and Radiotherapy, University of Munich, FRG*
*[3]Current address: Dept. of Surgery, University of Hansing, FRG*

*(Received 8 January 1991)*

The only curative treatment for proximal bile duct cancer with involvement of both main hepatic ducts is liver transplantation. Most patients do not fulfill the requirements for liver transplantation. Our treatment strategy in appropriate cases is palliative tumor resection and reconstruction of the biliary passage by sutureless bilioenteric anastomosis. We have treated 12 patients, 5 in combination with intraluminal and percutaneous radiotherapy. Our results indicate that this strategy leads to effective palliation in some cases provided that only microscopic residual tumor is left *in-situ*. Our survival times compare favourably with survival after liver transplantation.

KEY WORDS: Proximal bile duct cancer, palliation, intrahepatic sutureless bilioenteric anastomosis, intraluminal radiotherapy

### INTRODUCTION

Bile duct cancer is a rare tumor accounting for 7% of all gastrointestinal malignancies[1]. According to its location it can be subdivided into distal, intermediate and proximal tumors[2]. This classification is essential as treatment policy depends on tumor location.

In the past proximal bile duct tumors were classified into three types, according to their extension in relation to the hepatic confluence[3]. Type I are tumors of the proximal main hepatic duct, which have not reached the hepatic confluence. Tumors of the hepatic duct which have involved the hepatic confluence belong to type II. Type III is present if there is evidence of invasion of at least one main hepatic duct up to the branching of second order bile ducts. Due to therapeutic

Address correspondence to: Prov. Dos. Dr. med. Jakob R. Izbicki, Chururgische Klinik, Universitat Hamburg, Martiuistr. 52, D-2000 Hamburg 20, Germany, der Universität München, Nuβbaumstr. 20, D-8000 München 2, Germany

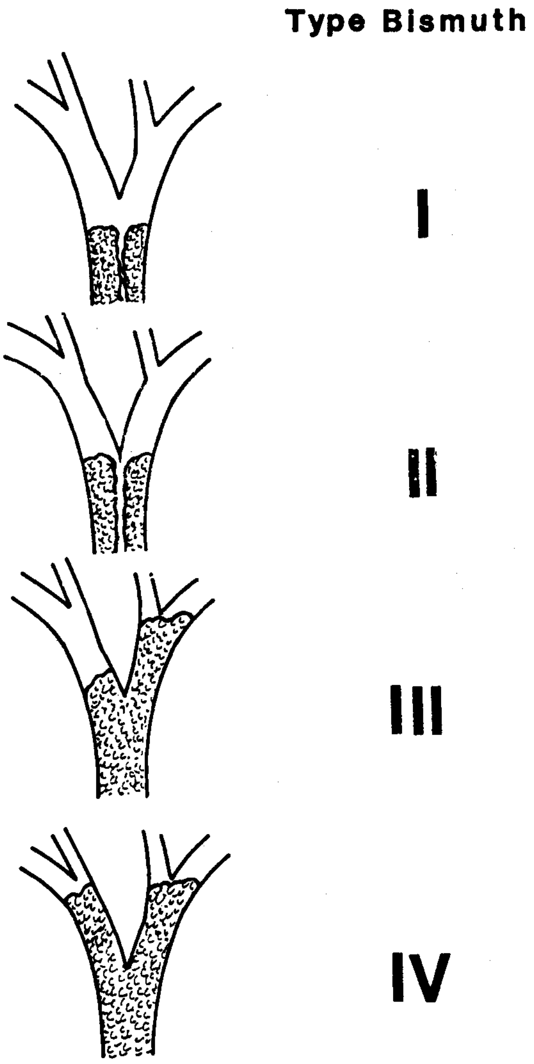

**Figure 1** Classification of proximal bile duct cancer Bismuth 1988[4].

considerations it is deemed useful to add a fourth type of proximal bile duct tumor which is characterized by invasion of both main hepatic ducts[4] (Figure 1).

A prerequsite for potentially curative resection of proximal bile duct cancer is that major structures of the porta hepatis like the portal vein and the hepatic artery are, either, not invaded by the tumor or that their continuity can be reconstructed after partial resection[5,6]. If this criterium is fulfilled, potentially curative resection is possible with a safety margin of at least 1 cm. In case of type I and II tumors curative treatment is based on local resection of the tumor combined with a radical lymphadenectomy in the hepatoduodenal ligament[6]. In our opinion, type I and II

tumors should be summarized in one category as the necessity of a safety margin of 1 cm will result in the same therapeutic regimen namely the resection of the hepatic confluence. The biliary passage is reconstructed by two or three hepaticojejunal anastomoses draining in one Roux-en-Y-loop of jejunum. If the tumor invades the second order bile duct branches of one side of the liver, resectional treatment with curative intention is possible by local resection of the tumor combined with a hemihepatectomy of the affected lobe and a lymphadenectomy. In order to achieve a sufficient margin of safety, it is necessary to resect the hepatic duct of the unaffected lobe of the liver[6]. The bilioenteric continuity is then reconstituted by a hepaticojejunal anastomosis using a Roux-en-Y-loop of jejunum. The caudate lobe drains by small bile duct branches into the right as well as the left hepatic duct. As hilar cancer spreads predominantly by continuity, the routine removal of the caudate lobe has been suggested in all type III cancers in order to achieve sufficient safety margins[7]. However, caudate lobe excision has so far not been accepted as a routine procedure[6]. Using this aggressive therapeutic approach to proximal bile duct cancer, median and mean survival times of 25 and 29 months are reported, respectively with reasonable morbidity and mortality rates[6].

If the tumor invades both main hepatic ducts up to the 2nd order bile duct branches, no form of hepatic resection can achieve adequate safety margins and hemihepatectomy is not indicated, as it would mean an unnecessary sacrifice of vital liver tissue in view of the questionable radicality.

Four therapeutic options must be considered for this type of tumor:

1. Liver transplantation[8,9].
2. Palliative tumor resection combined with radiotherapy[10,11].
3. Palliative decompression by percutaneous external drains or internal stents which can be placed either by percutaneous transhepatic or endoscopic access[12,13].
4. Surgical bypass by intrahepatic cholangioenteric anastomosis[14,15].
5. Radiotherapy by external and/or intraluminal irradiation[16–19].

We report on our experience with the second treatment modality in patients with Bismuth type IV proximal bile duct cancer.

*Patients*

Between August 1983 and June 1988 a total of 12 patients with proximal bile duct tumors involving both main hepatic ducts, Bismuth type IV (Figure 6a), were operated in the Department of Surgery, University of Munich. Five patients were men and 7 women. Ages ranged from 55 to 82 years and averaged 67 years (Table 1). The complete diagnostic work-up included computerized axial tomography, abdominal ultrasonography, digital subtraction angiography and endoscopic or percutaneous cholangiography with or without preoperative drainage in all patients.

*Operative Technique*

In all patients gross invasion of the hilar vessels was excluded by CAT-scan and

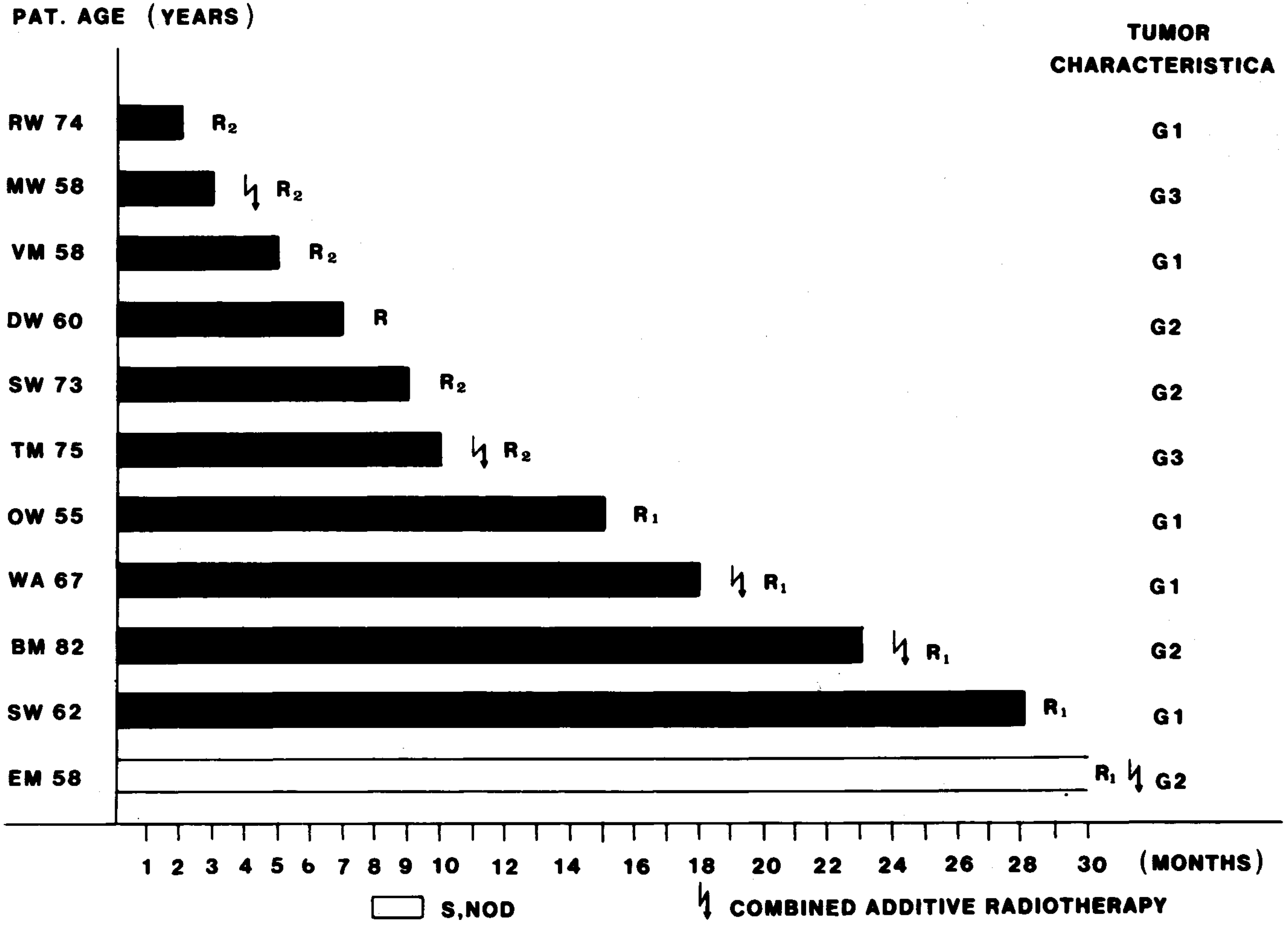

**Table 1** Patient characteristics and survival times digital subtraction angiography. No evidence of systemic spread was found preoperatively. All patients had resection of gross tumor involvement of the main hepatic ducts up to the level of the second order confluence. Our technique of tumor resection is based on the technique described by Cameron *et al.* 1982[11]. We also transsected the common bile duct at an early stage in the operation to facilitate clearance of the tumor from the portal vein. In three of these patients, tumor invasion of the gall bladder necessitated an additional partial resection of segment IV of the liver. Biliary drainage was ensured by constructing a bilioenteric anastomosis as described by R. Smith[20] (Figure 2). After resection of the tumor by an atypical resection of the hilar segments, 3–6 stumps of intrahepatic 2nd order bile ducts are exposed in the depth of the hepatic hilum. The segmental duct of segment V is cannulated via the common duct of segment V and VIII (depending on the depth of the resection) and a silicon tube (21 Charriere Robinson tube, Boehringer Ingelheim, FRG) is passed transhepatically to the antero-lateral surface of the liver. On the left side the segmental duct of segment III is cannulated via

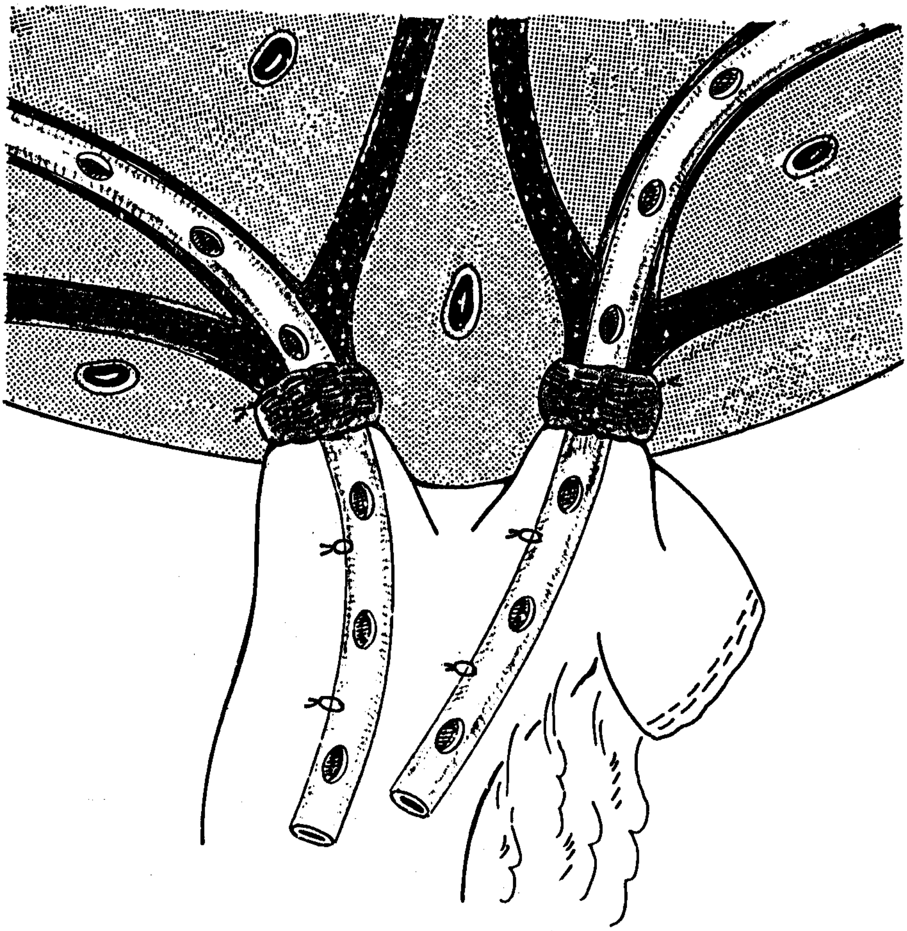

**Figure 2** Schematic drawing of the sutureless bilioenteric anastomosis Rodney Smith[17].

the common duct of segment II and III and a second silicon tube is advanced transhepatically. The right tube is advanced through the abdominal wall in the right hypochondrium, whereas the left tube is placed in the left epigastric region. Kinking has to be avoided at this stage. A Roux-en-Y loop of 30 cm in length is constructed and brought up via a retrocolic route to the hilum of the liver. On two neighbouring spots laying antimesenterically, a disc of seromuscular layer, diameter 1.5 to 2 cm is excised. The protruding mucosa is incised and a purse string suture is applied. The tubes are now passed into the bowel lumen. The tubes are secured by tying the purse string sutures. Two additional sutures (all suture material used is absorbable) are passed through the full thickness of the bowel wall adjacent to the mucosa and passed through the tube itself. They serve to fix the bowel to the tubes, when traction is applied (Figure 3). The jejunal loop is then brought up to the hepatic hilum by pulling on the tubes with slight tension and the tubes are fixed to the abdominal wall. Thus a sutureless bilioenteric anastomosis is created. The tubes are removed after 6 to 12 weeks (Figures 6a, 6b). Five patients received postoperative combined radiotherapy starting at three weeks postoperatively. A suitable length of 192-Iridium-wire, assessed from the cholangiographic appearance was placed within an angiographic guide wire. Under radiographic control the guide wire was positioned via the external limbs of the percutaneous transhepatic tubes until the segment of the active irridium lay within the main tumor bed (Figure 4). The radiation, calculated at 0.5 cm from the wire, was then

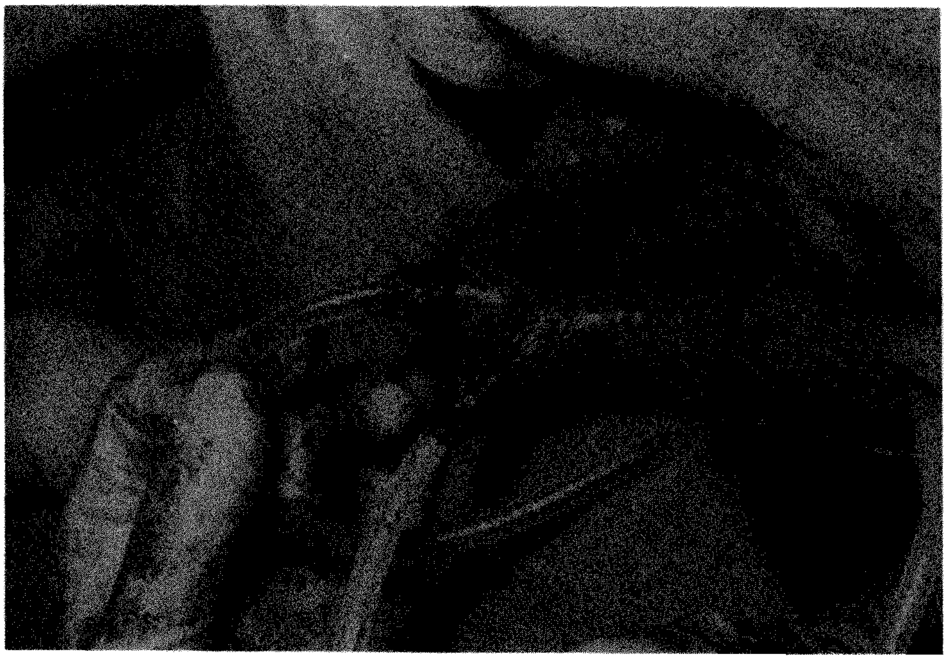

**Figure 3** Positioning of transhepatic tubes into the jejunal lumen.

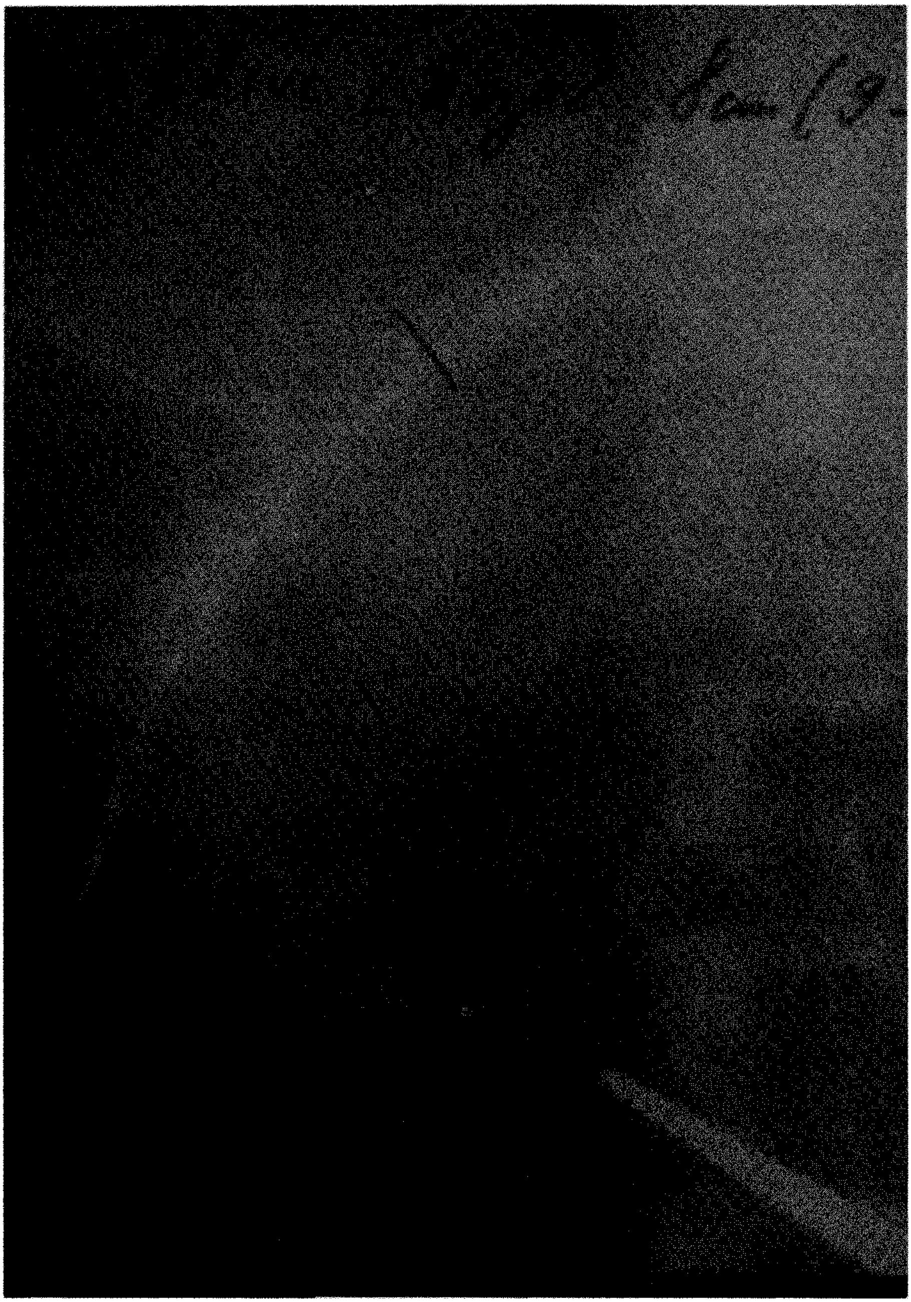

**Figure 4** Positioning of the Iridium-wire via the external limb of the transhepatic tubes into the tumor bed for intraluminal radiotherapy.

administered at a dose of 10 gray on three occasions at weekly intervals. Additionally external beam irradiation using a standard three field technique was given to the patients, so as to deliver 36 Gy. A total dose of 66 Gy was delivered. Radiation therapy was discontinued 7–8 weeks postoperatively.

### RESULTS

Preoperative risk assessment revealed severe operative risk factors in 9 patients, 5 patients showed more than one risk factor. In all 9 patients severe cardiovascular disease was present. Additionally two patients exhibited severe diabetes mellitus and one patient showed concommitant pulmonary or renal disease, respectively. Thus, more than two thirds of our patients were assessed as high risk patients, classified as class III according to the American Society of Anesthesiologists' Physical status classification[21]. In 6 patients the tumor was technically resectable and there was no evidence of gross residual tumor postoperatively. Pathohistological evaluation of the resected specimen confirmed microscopic residual tumor. In the remaining 6 patients gross residual tumor was evident after resection. These patients did not survive many months (Table 1). Three patients showed invasion of the gallbladder requiring resection of segment IV of the liver. In four patients nodal involvement of metastatic spread was evident at the time of surgery (Table 1). One 82 year old patient died of cardiac failure on the 7th postoperative day, otherwise there was no postoperative mortality. One patient developed an abscess of the liver, requiring percutaneous drainage and antibiotic therapy. In another patient a bile leak between liver surface and abdominal wall developed, which was drained and sealed spontaneously. In 3 early patients the tubes dislocated 2 weeks postoperatively without any clinical consequences. Two patients developed mild cholangitis. They were successfully managed with antibiotics. In two patients abdominal wound infections were evident. Thus, no life-threatening complications were observed.

Following discharge a continuous decline of serum bilirubin to normal levels was observed in 7 patients. In two patients with gross residual tumor a postoperative increase of serum bilirubin was observed. Both patients died of disease 2 and 3 months later, respectively. Two patients with gross residual tumor exhibited a delayed decrease in serum bilirubin, they survived 5 and 7 months, respectively (Figure 5). Ten patients died of disease in the observation period (Table 1). The mean survival time at this writing is 11.5 months, the median survival time is 10 months. In the one surviving patient serum bilirubin is still in normal range. Five of 11 patients survived for more than 12 months. The median symptomfree interval was 9 months. In this time the patients were able to lead a normal life without external tubes.

### DISCUSSION

Adequate treatment of Bismuth type IV proximal bile duct cancer requires subtle preoperative staging by ERC, Angiography and CAT-scan[4,5]. If distant metastases are evident, the survival time will be short. Therefore only a palliative procedure carrying minimal risk for the patient but which must effectively control jaundice

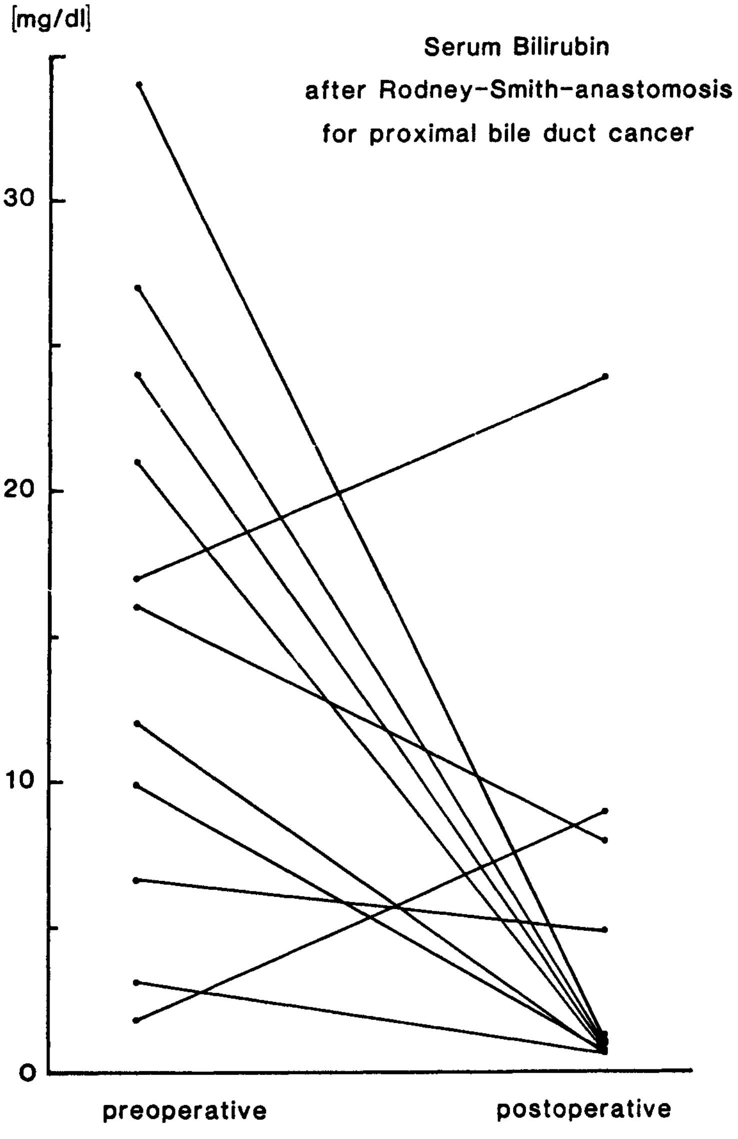

**Figure 5** Total serum bilirubin level in 11 patients after palliative tumor resection and sutureless bilioenteric anastomosis.

and pruritus is justified. In this case (systemic disease or local irresectability) endoscopic or radiologic palliative procedures are indicated[12,13,14]. These procedures include biliary prostheses, which can be introduced by endoscopic[13] or by percutaneous transhepatic means[12,14]. The endoscopic route seems to be associated with few complications,[18] but in most instances the percutaneous route is chosen.

External percutaneous transhepatic drainage (EPTD) is indicated if transtubation of the tumor by a biliary prosthesis is not possible because of the firmness of the tumor[12,13,14]. These methods are associated with limitations of quality of life and a high complication rate of up to 30% (cholangitis, catheter obstruction);[6,12,13,18,19] therefore they should be applied only to patients who pose severe symptoms of obstructive jaundice like pruritus or cholangitis and who have a very limited prognosis.

In case of locally advanced tumors surgical drainage procedures are reported in the literature. Palliation is achieved by creating a surgical bypass through intrahepatic cholangioenteric anastomosis with the tumor in the porta hepatis left behind[2,3,15]. These operations include the Longmire procedure and intrahepatic cholangioenteric anastomosis, in which the intrahepatic bile duct of segment III or V, respectively is joined to a Roux-en-Y-loop of jejunum after partial resection of the respective segment. A mortality rate of up to 10% is reported; therefore these procedures should be only performed in patients with acceptable operative risk. On the other hand, their palliative effect is only limited[6,11]. After adequate selection of patients the experience of some authors has been reported as excellent[3,4].

The only curative approach to hilar cancer with involvement of both main hepatic ducts is liver transplantation. Only patients, who

1. have no evidence of distant metastases
2. or regional lymphnodes
3. are under 60 years of age
4. have no severe concommitant disease should be selected for this procedure[6].

These selection criteria almost always lead to exclusion of patients with bile duct cancer, since the vast majority of patients presenting with this disease are over 60 years of age[22]. Therefore the appropriate surgical management of advanced proximal bile duct tumors is still not firmly established. None of our patients would have been a candidate for liver transplantation.

Our therapeutic concept of palliative tumor resection and additive combined radiotherapy is based on the idea that a radical resection of tumors which involve both main hepatic ducts is not possible for anatomical reasons. Residual disease is treated by radiotherapy[10,16,23]. This concept has been already described by Langer *et al.* and Cameron *et al.*[10,11], but mostly for tumors which involve the hepatic confluence and do not invade the level of the second order bile ducts. In Cameron's series[11] a mean survival time of 18 months was reported which seems to be more favorable than our experience with a mean survival time of 11.5 months. This difference could be explained by the fact, that 37% of patients in Cameron's series were resected with a curative intent, whereas in our series the localisation of the tumor was so unfavorable that only a palliative tumor resection could be carried out. Involvement of the resection margins by bile duct cancer is supposed to have a negative effect on survival,[10] but this issue has been questioned in a recent study[6].

In our experience, the palliation is especially favourable in patients where no gross residual disease is evident (R-1-resection). Patients with gross residual tumor had a short survival time and only limited benefit from the procedure. In order to achieve a R-1-stage of resection, the bile ducts have to be transected at the level of the 2nd order branches. Reconstruction of bilio-enteric continuity at this height meets technical problems. Basically there are two alternatives: A sutured cholangioenteric anastomosis[10,11] or a sutureless cholangioenteric anastomosis in the technique of Rodney Smith[20].

For technical reasons the first alternative requires resection of segments IV and V of the liver. In our view this means an unnecessary extension of the operation, especially if one considers the palliative character of the operation.

The sutureless cholangioenteric anastomosis is easy and safe to perform. Originally it was described for benign strictures of the proximal bile duct[20]. Our experience shows that it can be safely employed in malignant strictures, too. This type of cholangioenteric anastomosis is strongly opposed by some authors, who argue that only particular segments of the liver allow adequate biliary drainage[22]. In our experience the formerly undrained 2nd order bile duct branches gain access to the Roux-en-Y-loop (Figure 6b), as assessed by postoperative cholangiography via the tubes and regular ultrasound examinations. Moreover, it is easy to perform.

The cholostasis will be effectively controlled in most cases after palliative resection and cholangioenteric anastomosis (Figure 5). Besides its technical simplicity, this procedure offers the advantage of an easy access for intraluminal radiation therapy via the large percutaneous transhepatic tubes (Figure 3). In the case of a sutured anastomosis, most often several thin biliary stents are introduced, which do not provide a lumen wide enough for introduction of the iridium wire.

Bile duct cancer can be considered as a radiosensitive tumor[16,18,19,23]. In patients, treated by palliative drainage procedures with the tumors left in place, radiation therapy was able to induce prolongation of the average survival time[16]. In addition, regressive changes have been observed in irridiated tumors[25]. Intraluminal radiotherapy with 192-Iridium led to prolongation of survival as well; nearly 50% of patients survived for more than 12 months[19,24]. This figure is surprisingly high in view of our results, but the fact has to be considered that in these series bile duct tumors of all locations were included, whereas our series was comprised of patients with a particularly dismal prognosis due to advanced tumors involving both lobes of the liver. So far, the question whether palliative resection of the tumor or radiotherapy alone will lead to better palliation, cannot be answered. A prospective controlled clinical randomized trial is necessary for further evaluation.

A major set back to palliative biliary decompression combined with internal radiation therapy is the substantial mortality and morbidity, especially the high incidence of cholangitis, reaching a rate of up to 40%[19]. However, in a recent study, a mortality of zero and an incidence of cholangitis of 14% were described, with a median survival of 10.5 months[18].

Our approach to treatment of proximal bile duct cancer combines the advantages of intraluminal and percutaneous radiation therapy with improvement of quality of life, which results from palliative tumor resection. Due to our limited number of patients so far we cannot prove that additive radiotherapy indeed leads to prolongation of survival. The comparison of our approach to liver transplantation shows that our patients presented with a more unfavorable prognosis due to advanced age and concommitant risk factors. Nonetheless, the average survival time of our series was 11.5 months (median 10 months) as compared to a recent

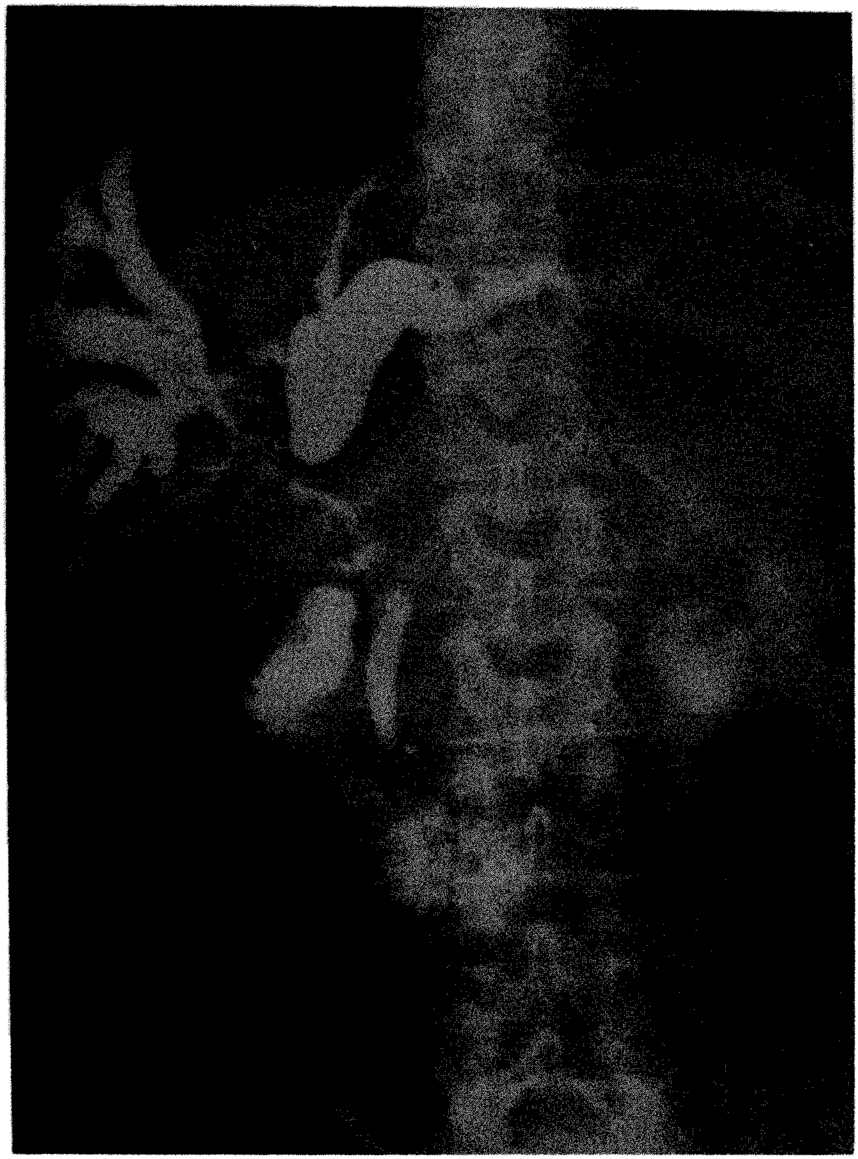

**Figure 6a** Pat. S.W. female, 62 years old. Preoperative percutaneous transhepatic cholangiography showing a proximal bile duct cancer with invasion of both main hepatic ducts and extension to the 2nd order bile ducts.

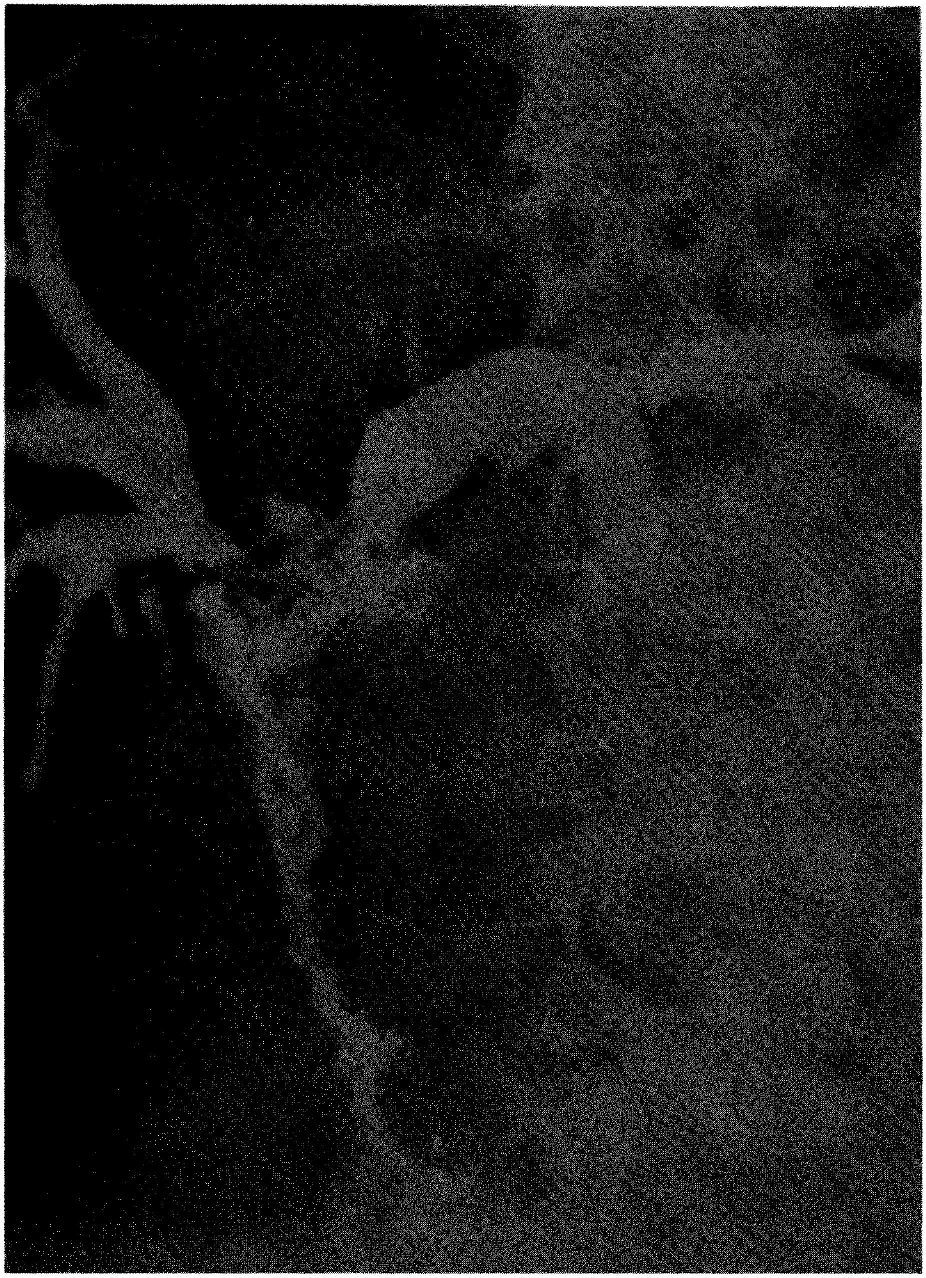

**Figure 6b** Pat. S.W. female, 62 years old. Cholangiography 4 weeks after palliative tumor resection and bilateral sutureless bilioenteric anastomosis. The intrahepatic bile ducts are draining into the Roux-en-Y-loop.

series of 16 patients after liver transplantation (average survival time 9.5 months, median 16 months)[8]. Moreover, 6–8 weeks postoperatively the treatment is terminated, the patient is devoid of tubes and a complex follow-up as after liver transplantation is not necessary.

Our therapeutic approach leads to effective palliation with acceptable morbidity and mortality if one succeeds in resecting the tumor without leaving gross residual tumor behind.

### *References*

1. Tompkins, R.K., Thomas, D., Wile, A. and Longmire, W.P. Jr. (1981) Prognostic factors in bile duct carcinoma. *Ann. Surg.*, **194**, 447–457
2. Longmire, W.P. jr., McArthur, M.S., Bastounis, E.A. and Hiatt, J. (1973) Carcinoma of the extrahepatic biliary tract. *Ann. Surg.*, **178**, 333–345
3. Bismuth, H., Corlette, M.B. (1975) Intrahepatic Cholangioenteric Anastomosis in Carcinoma of the Hilus of the Liver. *Surg. Gynecol. Obstetr.*, **140**, 170–178
4. Bismuth, H., Castaing, D. and Traynor, O. (1988) Resection or Palliation: Priority of Surgery in the Treatment of Hilar Cancer. *World J. Surg.*, **12**, 39–47
5. Lygidakis, N.J. (1987) Chirurgische Behandlung des Karzinoms des Leberhilus: M<ou> glichkeiten der radikalen Resektion. In: Schumpelick V., Pichlmayr R., *Chirurgie der Leber*, pp 152–164. Springer-Verlag: Berlin, Heidelberg
6. Hadjis, N.S., Blenkharn, J.I., Alexander, N., Benjamin, I.S. and Blumgart, L.H. (1990) Outcome of radical surgery in hilar cholangiocarcinoma. *Surgery*, **107**, 597–604
7. Mizumoto, R., Kawarada, Y. and Suzuki, H. (1986) Surgical Treatment of Hilar Carcinoma of the Bile Duct Surgery. *Gynecology & Obstetrics*, **162**, 153–158
8. Pichlmayr, R., Ringe, B., Lauchart, W., Bechstein, W.O., Gubernatis, R. and Wagner, E. (1988) Radical Resection and Liver Grafting as the Two Main. Components of Surgical Strategy in the Treatment of Proximal Bile Duct Cancer. *World J. Surg.*, **12**, 68–77
9. Iwatsuki, S., Gordon, R.D., Shaw, B.W. and Starzl, T.E. (1985) Role of Liver Transplantation in Cancer Therapy. *Ann. Surg.*, **202**, 401–407
10. Langer, J.C., Langer, B., Taylor, B.R., Zeldin, R. and Cummings, B. (1985) Carcinoma of the extrahepatic bile ducts: Results of an aggressive surgical approach. *Surgery*, **98**, 752–759
11. Cameron, J.L., Broe, P. and Zuidema, G.D. (1982) Proximal bile duct tumors: Surgical management with silastic transhepatic biliary stents. *Ann. Surg.*, **196**, 412–419
12. Mueller, P.R., Van Sonnenberg, E. and Ferruci, J.T. jr. (1982) Percutaneous biliary drainage: Technical and catheter related problems in 200 procedures. *AJR*, **138**, 17–28
13. Cotton, P. (1984) Endoscopic methods for relief of malignant obstructive jaundice. *World J. Surg.*, **8**, 854–861
14. Nakayama, T., Ikeda, A. and Okuda, K. (1978) Percutaneous transhepatic drainage of the biliary tract. *Gastroenterology*, **74**, 554–559
15. Hepp, J., Moreaux, J. and Lechaux, J.P. (1973) Les anastomoses biliodigestives intrahepatiques dans les cancers des voies biliaires. Resultats de 62 interventiones. *Nouv Presse Med.*, **2**, 1829–1836
16. Johnson, D.W., Safai, C. and Goffinet, D.R. (1985) Malignant Obstructive Jaundice: Treatment with External-Beam and Intracavitary Radiotherapy. *Radiation Oncology Biol. Phys.*, **11**, 411–416
17. Chitwood, W.R., Meyers, W.C., Heaston, D.K., Herskovic, A.M., McLeod, M.E. and Jones, R.C. (1982) Diagnosis and treatment of primary extrahepatic bile duct tumors. *Ann. J. Surg.*, **143**, 99–106
18. Ede, R.J., Williams, S.J., Hatfield, A.R.W., McIntyre, S. and Mair, G. (1989) Endoscopic management of inoperable cholangiocarcinoma using Iridium-192. *Brit. J. Surg.*, **76**, 867–869
19. Karani, J., Fletcher, M., Brinkley, D., Dawson, J.L., Williams, R. and Nunnerly, H.C. (1985) Internal biliary drainage and local radiotherapy with Irdium-192 wire in treatment of hilar cholangiocarcinoma. *Clin. Radiol.*, **36**, 603–606
20. Smith, R. (1964) Hepaticojejunostomie with transhepatic intubation. *Brit. J. Surg.*, **51**, 186–194
21. Hallen, B. (1973) The American Society of Anesthesiologists' Physical Status Classification. *Acta Anaesthet. Scand.*, **52** (Suppl.) 5

22. Blumgart, L.H. and Kelley, C.J. (1984) Hepaticojejunostomie in benign and malignant high bile duct strictures. Approaches for the left hepatic ducts. *Br. J. Surg.*, **71**, 257–262
23. Buskirk, S.J., Gunderson, L.L., Adson, M.A. *et al.* (1984) Analysis of Failure Following Curative Irradiation of Gallbladder and Extrahepatic Bile Duct Carcinoma. *Radiation Oncology Biol. Phys.*, **10**, 2013–2023
24. Fletcher, M.S., Brinkley, D., Dawson, J.L., Nunnerley, H. Williams, R. (1983) Treatment of hilar carcinoma by bile drainage combined with internal radiotherapy using 192-iridium wire. *Br. J. Surg.*, **70**, 733–735
25. Kutzner, J., Klose, K., Keller, E. (1985) Palliative Gallengangsbestrahlung. *Stahlentherapie*, **161**, 669–672

(*Accepted by S. Bengmark 28 October 1991*)

